# Little Helpers or Mean Rogue—Role of Microglia in Animal Models of Amyotrophic Lateral Sclerosis

**DOI:** 10.3390/ijms22030993

**Published:** 2021-01-20

**Authors:** Hilal Cihankaya, Carsten Theiss, Veronika Matschke

**Affiliations:** 1 Department of Cytology, Institute of Anatomy, Ruhr-University Bochum, D-44801 Bochum, Germany; hilalcihankaya@gmail.com (H.C.); carsten.theiss@rub.de (C.T.); 2 International Graduate School of Neuroscience (IGSN), Ruhr-University Bochum, D-44801 Bochum, Germany

**Keywords:** ALS, microglial activation, neuroinflammation, superoxide dismutase 1, chromosome 9 open reading frame 72, transactive response DNA binding protein 43, wobbler mouse

## Abstract

Amyotrophic lateral sclerosis (ALS) is one of the most common neurodegenerative diseases, causing degeneration of both upper and lower motor neurons in the central nervous system (CNS). ALS patients suffer from hyperreflexia, spasticity, paralysis and muscle atrophy and typically die due to respiratory failure 1–5 years after disease onset. In addition to the degeneration of motor neurons on the cellular level, ALS has been associated with neuroinflammation, such as microgliosis. Microglial activation in ALS can either be protective or degenerative to the neurons. Among others, mutations in superoxide dismutase 1 (SOD1), chromosome 9 open reading frame 72 (C9Orf72), transactive response DNA binding protein (TDP) 43 and vacuolar protein sorting-associated protein 54 (VPS54) genes have been associated with ALS. Here, we describe the dual role and functionality of microglia in four different in vivo ALS models and search for the lowest common denominator with respect to the role of microglia in the highly heterogeneous disease of ALS.

## 1. Introduction

Amyotrophic lateral sclerosis (ALS) is a fatal neurodegenerative disease which is characterized by rapid progressive degeneration of the upper motor neurons in the motor cortex and the lower motor neurons in the spinal cord and brain stem. The incidence of ALS is 2/100,000 in Europe, and older men are mostly affected, rather than women [1]. The most common form of ALS, seen in 90–95% of patients, is called sporadic ALS (sALS). To date, no genetic factors have been identified as being related to sALS. The second form of the disease is familial ALS (fALS), which is present only in 5–10% of the patients. Among others, mutations in superoxide dismutase 1 (SOD1), chromosome 9 open reading frame 72 (C9Orf72), transactive response DNA binding protein (TDP) 43 and fused in sarcoma (FUS) genes have been associated mostly with fALS [2]. Thirdly, there is a very rare form of ALS, known as juvenile ALS (jALS). ALS patients suffer from hyperreflexia, spasticity, paralysis, muscle weakness and muscle atrophy [3]. This progressive disease causes death due to respiratory failure 1–5 years after the onset of disease symptoms [3]. On the cellular level, ALS has been associated with motor neuron degeneration, neuroinflammation, increased oxidative stress, mitochondrial dysfunction, protein aggregation, impaired RNA processing, as well as impaired axonal transport [1]. A growing body of evidence suggests that not only motor neurons, but also glial cells like microglia are involved in ALS pathogenesis [4].

Microglia are the resident immune cells, and they comprise approximately 20% of the glial cell population in the central nervous system (CNS) [5]. Resting microglia have ramified morphology to survey the danger signals in the CNS parenchyma. Quiescent microglia transform into an amoeboid phenotype to become activated upon CNS diseases, injuries or pathogens. The morphology of microglia can be studied using specific markers, such as ionized calcium binding adaptor protein 1 (IBA1) or cluster of differentiation 11b molecule (CD11b) independently of their activation status. For example, IBA1, one of the microglia/macrophage markers and a calcium-binding protein [6], is well-studied in ALS animal models to demonstrate the cellular morphology of microglia and microgliosis, depending on the number and/or size of the IBA1 stained cells in the tissue. CD11b is an alpha integrin, which combines with CD18 to form a microglia/macrophage receptor on the cell membrane and plays a role in cell adhesion, migration, phagocytosis and survival at the inflammation site [7]. Additionally, other clusters of differentiation molecules are also utilized to characterize the microglia such as CD36 and CD68, which are activated by toll-like receptor (TLR) signaling [8,9], and CD40, which is required for microglia activation [10].

The morphology of microglia in ALS animal models varies depending on genetic background and the investigated region of the CNS (Table 1). It should be mentioned that both symptomatic and non-symptomatic animals of different models show predominantly amoeboid, and thus activated, microglia in the spinal cord and motor cortex.

**Figure 1:**
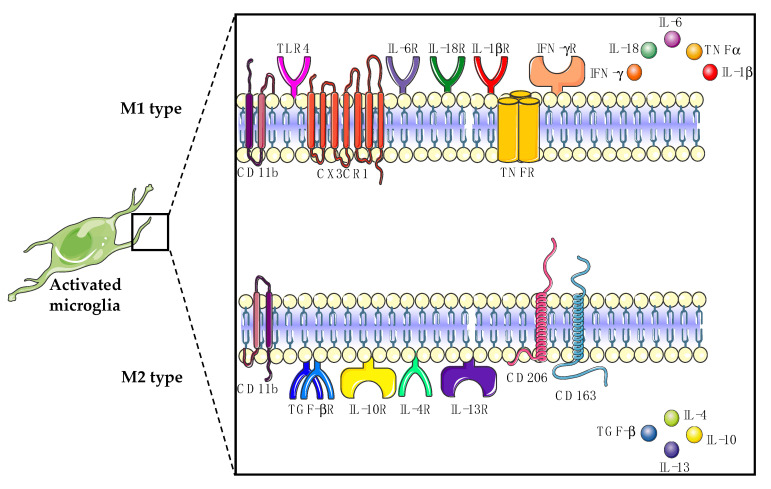
Classically activated M1-type microglia release proinflammatory cytokines, whereas alternatively activated M2-type microglia release anti-inflammatory cytokines. CD: cluster of differentiation; CX3CR1: C-X3-C motif chemokine receptor 1; TNFα: tumor necrosis factor alpha; TNFR: TNFα receptor; IFN-γ: interferon gamma; IFN-γR: IFN-γ receptor; TLR4: toll-like receptor 4; IL: interleukin; TGFβ: transforming growth factor beta; TGFβR: TGFβ receptor.

Nowadays, it is known that the functional differentiation of microglia based on expression patterns, phenotype, and stimulus is far more complex than the concept of “classically activated” M1 and “alternatively activated” M2 polarization of microglia (Figure 1) [49]. In this review, we make use of this oversimplified microglial classification, since most studies in the field of ALS still use this categorization. M1-type microglia have been shown to contribute to motor neuron degeneration in ALS by releasing proinflammatory cytokines such as tumor necrosis factor alpha (TNFα), interleukin 1 beta (IL-1β), interferon gamma (IFN-γ) and chemokines such as reactive oxygen species (ROS) and nitric oxide synthase (NOS) to initiate the immune response [4,50]. On the other hand, M2-type microglia can enhance the motor neuron survival in ALS by releasing anti-inflammatory cytokines such as transforming growth factor beta (TGFβ), interleukin 4 (IL-4), interleukin 10 (IL-10), interleukin 13 (IL-13) and neurotrophins such as insulin-like growth factor (IGF-1) and brain-derived neurotrophic factor (BDNF) to repair and restore the damaged tissue [4,50].

Therefore, it is assumed that activated microglia play a dual role during the pathogenesis of ALS. It was hypothesized that M2-type microglia could be protective for the motor neurons during the early stage of ALS. However, as the disease progresses, a shift from neuroprotection to neurotoxicity has been noted, where M1-type microglia induce neuroinflammation and motor neuron degeneration at the later stages of ALS [51]. Thus, neuroinflammatory processes such as microgliosis seem to play a major role in the ALS pathology [52,53,54].

More recently, together with the identification of ALS-linked genes, the number of mouse models for ALS created by genetic engineering methods has increased significantly. Thus, various models are now available for the investigation of the pathological mechanisms in ALS. These mouse models show microgliosis in CNS tissue, similarly to ALS patients. It was demonstrated that proinflammatory and anti-inflammatory molecules are released by M1- and/or M2-type microglia, leading to modulation of the microglial activation states in ALS [55,56,57]. In various animal models of ALS, the application of different compounds has been able to improve the survival of motor neurons. Thereby, different targets of ALS pathology have been addressed, such as hyperexcitability and excitotoxicity, neuroinflammation and oxidative stress. Two compounds, riluzole and edaravone, which reduce excitotoxicity and oxidative stress, respectively, have already been approved for ALS patients, but with only moderate success. Other agents that interfere with immune response mechanisms and thus target neuroinflammation, such as TLR-4 inhibitors, H1-receptor agonists and glucocorticoid receptor modulators, also showed a positive outcome on motor neuron survival in ALS animal models. Nevertheless, they remained rather unsuccessful after translation into clinical trials [58]. Reasons for failure could include the following: (i) preclinical studies have mostly been performed in only one ALS animal model, so the strong heterogeneity of the causes of the disease in different ALS cohorts would not be taken into account; (ii) an appropriate time window for the successful use of agents cannot be maintained in human patients [58]. The heterogeneity of the disease’s etiology thus requires a target that addresses as many patient groups as possible. We thus compare in this review the behavior, properties and functions of microglia in the different animal models to get closer to the goal of a common target.

## 2. SOD1 Model

About 25 years ago, the first gene mutation in the human superoxide dismutase (SOD) 1 gene was linked to the disease of ALS [59]. However, in Europe only 15% of fALS and 1–2% of sALS cases have been associated with mutations in the *SOD1* gene [60]. *SOD1* encodes an enzyme responsible for the detoxification of free superoxide radicals by converting them to hydrogen peroxide and molecular oxygen. However, a mutated allele can acquire both gain-of-function and loss-of-function mutations. It is currently assumed that toxic gain-of-function mutations in the *SOD1* gene generate mutation-induced misfolding of SOD1 and subsequent SOD1 aggregation [61]. This results in increased oxidative activity, leading to redundant production of hydrogen peroxide and molecular oxygen, together with an increased number of protein–protein interactions [62]. These days, more than 170 mutations have been described in human ALS cases (http://www.hgmd.org, accessed in January 2021) and numerous SOD1 animal models have been developed. Based on the genetic background, the type of mutation and the copy number of the mutated *SOD1* allele, animals can vary in showing ALS symptoms with different progression rates [63,64,65,66]. In the case of the most studied mutation—that causing a glycine-to-alanine change at the 93rd codon (G93A) in the *SOD1* gene—animals carrying mutant alleles, depending on the genetic background, have a range of survival of 17 to 26 weeks, and the disease onset starts about the 13th–17th weeks of age, when animals start to develop hind limb weakness [63,64,66,67,68]. One to two months after the disease onset, which is also called the symptomatic stage, *SOD1^G93A^* mice begin to show tremors, locomotor deficits, paralysis, mitochondrial vacuolization, neurofilament aggregations, gliosis and degeneration of axons and motor neurons [67]. This ALS model, as well as models with other *SOD1* mutations underlying ALS, shows microglial changes predominantly in the disease onset and symptomatic phase in different regions of the CNS, as summarized in the following paragraphs and in Table 2.

### 2.1. Microglial Morphology of the SOD1 Models

It is known that *SOD1^G93A^* mice show increased microgliosis, as a significant increase in IBA1 in Western blot analysis was found in the spinal cords of the pre-symptomatic and symptomatic *SOD1^G93A^* mice [11]. Medial reticular formation of *SOD1^G93A^* mice showed IBA1+ activated microglia, which were found in close proximity to Chx10+ glutamatergic V2a neurons [15]. In 10- and 14-week-old *SOD1^G93A^* mice, mRNA expression of *Iba1* was increased in the spinal cord when compared to *WT-SOD1* mice^13^. A total of 96% of the IBA1 signal from the spinal cord of *SOD1^G93A^* mice showed colocalization with keratan sulfate marker 5D4 [69]. There were no significant changes observed between wild-type and end-stage *SOD1^G93A^* mice in terms of the number of IBA1+ microglia taken from the external plexiform layer of the olfactory bulb and the internal plexiform layer of the retina [17]. The numerical density of IBA1+ and pSTAT3+/IBA1+ microglia gradually increased from 9 weeks to 21 weeks of age in the ventral spinal cords of *SOD1^G93A^* mice and most of the pSTAT3+/IBA1+ microglia were also positive for proinflammatory microglial markers Kv1.3 and Kv1.5 in 21-week-old *SOD1^G93A^* mice [70]. The increased number of IBA1+ cells were reduced in the cervical spinal cord and in the motor cortex of transgenic Klotho-overexpressing 16-week-old *SOD1^G93A^* mice (Klotho is an anti-inflammatory, antioxidative, neuroprotective and promyelinating protein.) [26]. Ubiquitin-positive aggregations surrounded by IBA1+ microglia were observed in both *SOD1^His46Arg^* and *SOD1^His46Arg^SQSTM1* (SQSTM1: sequestosome 1 is an adaptor protein regulating autophagy and anti-oxidative stress pathway) mice; however, there were no statistical differences between these groups [71]. *Iba1* and *CD68* mRNA levels remained unchanged, when NOX2 was knocked-out in *SOD1^G93A^* mice [19]. Broad spectrum NOX inhibitor perphenazine treatment of *SOD1^G93A^* mice increased *Iba1* and *CD68*, whereas administration of another NOX inhibitor, thioridazine, resulted in a significant decrease of *Iba1* and *CD68* in the spinal cord of *SOD1^G93A^* mice [19]. Ablation of serotonin 2B receptor (5-HT_2b_) in *SOD1^G86R^* mice resulted in a reduced IBA1 immunoreactivity in the spinal cord, where almost 50% of IBA1+ cells displayed cytoplasmic fragmentation [27].

In detail, both female and male *SOD1^G93A^* mice showed increased IBA+ immunostaining in the ventral horn when compared to WT litters; however, long term interleukin 33 (IL-33) treatment of *SOD1^G93A^* mice failed to alter microgliosis in either gender [25]. IBA1 quantification of tempol (a cyclic nitroxide)-injected *SOD1^G93A^* mice at 14 weeks (early symptomatic phase) showed less microglial activation in the ventral horn of the spinal cord, and tempol-injected *SOD1^G93A^* mice at 20 weeks (end stage) revealed a significant reduction in the microglial activity in the ventral, intermediate and dorsal regions of the spinal cord, when compared to the vehicle and riluzole-treated groups, respectively [14]. In different studies, Gamisoyo-San (a herbal medicine), TRAM-34 (selective Ca^2+^ activated K^+^ channel KCa3.1 inhibitor), TAK-242 (exogenous synthetic selective inhibitor of TLR4) and KCHO-1 (a herbal combination compound containing 30% ethanol and nine different herbal extracts) administration to *SOD1^G93A^* mice attenuated the IBA1 expression in the spinal cord [18,23,28,29]. A tyrosine kinase inhibitor (masitinib) treatment of post-paralyzed *SOD1^G93A^* rats ameliorated the microgliosis in the ventral spinal cord, as shown by IBA1 immunostaining [72]. Infusion of 15-week-old *SOD1^G93A^* brains with antisense oligonucleotide against cytosolic phospholipase A_2_ alpha (cPLA_2_α) inhibited the microglial activation in the spinal cord sections, as detected by IBA1 and CD40 immunostaining [21]. Immunohistochemical staining of AMD3100 (an inhibitor of CXCL12 binding to CXCR4)-treated *SOD1^G93A^* mice revealed significantly reduced IBA1 and CD36 levels in the spinal cord [22].

Rather than IBA1, CD11b has also been used in studies to investigate microgliosis and the general morphology of the microglia. Symptomatic 22–24-week-old *NLRP3-GFP*-*SOD1^G93A^* mice demonstrated NLRP3-GFP colocalization with CD11b in the ventral lumbar spinal cord [73]. Brain-derived extracellular micro-vesicles (BDEVs) from 2- and 5-month-old *SOD1^G93A^* mice revealed less than 5% CD11b+ vesicles in the flow cytometry analysis, indicating the absence of microglial markers in these BDEVs [74]. Although surprisingly decreased *CD11b* mRNA was detected in the spinal cord of the pre-symptomatic *SOD1^G93A^* mice, a significant increase was seen in the *CD11b* levels in the symptomatic *SOD1^G93A^* mice [11]. CD11b expression was significantly decreased in the spinal cord of the *SOD1^G93A^Grm5^+/−^* mice, when compared to *SOD1^G93A^* mice (Grm5: mGluR5 inactivating mutation) [16]. Gamisoyo-San (GSS) treatment of *SOD1^G93A^* mice reduced the CD11b expression in the spinal cord [28]. Treatment of *SOD1^G93A^* mice with IL-2c reduced CD11b immunoreactivity by 50% in spinal cord sections [20]. *SOD1^G93A^* mice that were either forced to exercise or treated with nandrolone (an anabolic androgenic steroid) in addition to physical exercise showed enhanced CD11b immune reactivity in the ventral horn of the lumbar spinal cord [75].

### 2.2. Microglial Markers in the SOD1 Models

Proinflammatory factors have been intensively investigated in several SOD1 models due to their cytotoxic effects on motor neuron degeneration. TLR2, TLR4, nuclear factor kappa B (NFκB) and high mobility group box protein 1 (HMGB1) protein levels remained unaltered in the spinal cord of the pre-symptomatic *SOD1^G93A^* mice; however, protein levels of TLR2 and TLR4 were decreased, together with an increase in the HMBG1, NFκB and phosphorylated NFκB levels in the spinal cord of the symptomatic *SOD1^G93A^* mice [11]. mRNAs of M1-type markers *MhcII*, *Cebpa* and *CD80* remained unchanged in the pre-symptomatic *SOD1^G93A^* mice; however, they were significantly increased in the symptomatic *SOD1^G93A^* mice [11]. CX3CR1, the only receptor of chemokine (C-X-C motif) ligand 1 (CX3CL1), was found to be located mainly in the microglia of *SOD1^G93A^* mice, as shown by IBA1 and CX3CR1 double immunofluorescent staining [76]. In the same study, the number of CD86+/IBA1+ microglia was significantly increased in the anterior horn of *SOD1^G93A^* mice at P90 and P120 [76]. Concordantly, M1-type markers *iNOS* and *CD86*, together with M1-type cytokines *Il-1β* and *Tnfα*, were increased at the mRNA level in P90 and P120 *SOD1^G93A^* mice [76]. HuR, an RNA binding protein, colocalized with IBA1 in the cytoplasm of activated microglia in spinal cord of 18-week-old mutant SOD1 mice [30]. Arylsulfatase B (ARSB) was found to be partially expressed by microglial cells in both wild-type and *SOD1^G93A^* mice [77]. Ablation of serotonin 2B receptor (5-HT_2b_) in *SOD1^G86R^* mice resulted in reduced mRNA levels of *Nox2*, *Ccl4* and *MhcII* in the brain stem [27].

Nucleotide binding domain and leucine rich repeat containing protein 3 (NLRP3) inflammasome is an intracellular multiprotein complex that can activate the innate immune system upon interaction with foreign molecules. This activation can trigger neuroinflammation, as observed in animal ALS models and ALS patients [73]. mRNA expressions of *Il-1β*, *Nlrp3*, *Asc* and *Caspase-1* were upregulated in the spinal cord samples of 22–24-week-old *SOD1^G93A^* mice [73]. *Nlrp3*, *Il-1β*, *Il-6* and *Tnfα* mRNAs were downregulated in pre-symptomatic *SOD1^G93A^* mice; however, *Nlrp3*, *Il-1β*, *Il-10* and *Tnfα* mRNAs were upregulated in symptomatic *SOD1^G93A^* mice [11]. *Nlrp3* and *Il-1β* mRNA expressions were induced in both 9-week-old and 14-week-old *SOD1^G93A^* mice, whereas *Asc* and *Il-18* mRNA expressions remained unchanged among wild-type and *SOD1^G93A^* groups [12]. Protein levels of ASC, NLRP3, IL-1β, IL-18 and active caspase 1 were found to be increased in *SOD1^G93A^* mice [12]. IBA1+ cells did not colocalized with NLRP3; however, ASC colocalized with both GFAP and IBA1 cells of the *SOD1^G93A^* mice [12].

To attenuate the neurodegenerative proinflammatory effects of microglia, several therapeutic substances have been used in different SOD1 models. Tempol treatment of *SOD1^G93A^* mice reduced the expressions of proinflammatory cytokines *Il-1β*, *Tnfα* and *iNos*, whereas *Ifnγ* expression did not show any statistical difference in the spinal cord samples [14]. Gamisoyo-San (GSS) treatment of *SOD1^G93A^* mice downregulated the TLR4 signaling related proteins TLR4, CD14 and COX-2 in the spinal cord [28]. Noggin (an antagonist of bone morphogenetic protein)-treated *SOD1^His46Arg^* transgenic rats showed no statistical difference in IBA1 staining of the spinal cord, when compared to controls, however Western blot and immunostaining of CD68-activated microglia showed less immunoreactivity in the noggin-administered lumbar spinal cords of *SOD1^His46Arg^* transgenic rats [78]. In the same study, triple immunostaining of IL-1β-CD68-GFAP, iNOS-CD68-GFAP and TNFα-CD68-GFAP proved that astrocytes predominantly express IL-1β, iNOS and TNFα, rather than activated microglia [78]. Furthermore, noggin treatment of *SOD1^His46Arg^* transgenic rats demonstrated significantly decreased *iNOS* and *TNFα* expressions both in astrocytes and microglia, whereas *Il-1β* expression particularly decreased in the astrocytes [78]. BMP4-targeted anti-sense oligonucleotide administration of *SOD1^His46Arg^* rats downregulated CD68-activated microglia; however, IBA1+ microglia were not affected by BMP4 suppression [78]. TAK-242 treatment of *SOD1^G93A^* mice decreased *Tnfα*, but not *Il-1β* mRNA levels in the spinal cord samples [29]. KCHO-1 treatment of *SOD1^G93A^* mice reduced iNOS expression in the spinal cord when compared to the vehicle-treated samples [18]. Short-term clemastine treatment of *SOD1^G93A^* mice insignificantly reduced CD68 and NFκB; however, long term clemastine (1st generation histamine H1R antagonist) treatment produced the opposite effects, when compared to the vehicle-treated mice [24]. AMD3100-treated *SOD1^G93A^* mice revealed significantly reduced TNFα and IL-6 levels in the spinal cord [22].

In addition to proinflammatory cytokines, the effects of anti-inflammatory factors have also been examined in SOD1 models. The number of Arg1+/IBA1+ microglia was significantly higher in the anterior horn of the P90 *SOD1^G93A^* mice; however, it was reduced at P120 [76]. Concordantly, mRNA levels of M2-type markers *Arg-1* and *CD206*, together with M2-type cytokine *Il-10*, were increased in P90, but declined in P120 *SOD1^G93A^* mice^76^. *Arg1*, *Fizz1*, *Socs1*, *Tgfβ1* and *CD206* mRNAs were found to be significantly decreased in the pre-symptomatic *SOD1^G93A^* mice; however, expressions of only *Arg1* and *Socs1* were reduced in the symptomatic *SOD1^G93A^* mice [11]. On the other hand, increased levels of *Tgfβ1* and *CD206* were detected in the symptomatic *SOD1^G93A^* mice [11]. Ablation of serotonin 2B receptor (5-HT_2b_) in *SOD1^G86R^* mice resulted in reduced mRNA levels of *Tgfβ1*, *TgfβR1*, *Ym1* and *Tyrobp* in the brain stem [27].

To boost the neuroprotective anti-inflammatory effects of microglia, several drug treatments have been carried out using SOD1 models. Increased gene expression levels of the neurotrophic factors *BDNF* and *GDNF* were shown to be decreased in tempol- and riluzole-treated 14-week-old *SOD1^G93A^* mice, respectively [14]. In contrast with the predominant assumption that TGFβ is an anti-inflammatory cytokine, it can also induce inflammation, acting as a proinflammatory agent [79]. Consequently, the *Tgfβ* mRNA level was reduced by tempol administration in *SOD1^G93A^* mice, whereas *Il-10* expression remained at the same level among vehicle-, riluzole- and tempol-treated groups [14]. Ninety-day-old *SOD1^G93A^* mice receiving total body irradiation together with intravenous transplantation of GFP+ donor bone marrow cells revealed an accumulation of bone-marrow-originated M2-type microglia in the ventral spinal cord, and this was attributed to the permeability in the blood brain barrier [80]. IL-33 treatment of *SOD1^G93A^* mice did not alter Arg-1 immunoreactivity in the female group; however, it increased the Arg-1 protein expression of the males in the ventral horn [25]. The amounts of Arg1 and CD163 after clemastine treatment of P120 *SOD1^G93A^* mice were increased by 2.5-fold and 3-fold, respectively, when compared to the wild-types [24]. Additionally, protein levels of purinergic receptors P2X7 and P2Y12 after clemastine treatment of P120 *SOD1^G93A^* mice were increased by 3-fold and 5-fold, respectively, when compared to the vehicle-treated mice, whereas the P2X4 level remained unchanged [24].

### 2.3. Transcriptome Profiling of the SOD1 Models

Single-cell RNA sequencing analysis of wild-type and mutant *SOD1* mice at P100 revealed 227 differentially expressed genes in the microglia of the brain stem, in which *Sod1* and *Camk2b* genes were upregulated and *Nav2*, *mt-Rnr2* and *1700112E06Rik* genes were downregulated [81]. TaqMan gene expression assays of SOD1- and Klotho-overexpressing *SOD1* mice revealed significant differences—*Il-1β*, *Il-12a* and *Tnfα* mRNAs were downregulated, whereas *Mag* mRNA was overexpressed in the motor cortex [26]. Furthermore, *Fzd5*, *Il-1α*, *Tnfaip2* and *Tnfα* mRNAs were significantly downregulated, whereas *Vegf*, *Mbp*, *Mag*, *Nrf2*, *Prx-3* and *Prx-2* mRNAs were upregulated in the lumbar spinal cord [26]. Spatial transcriptomics of L3 to L5 sections of the spinal cord from both wild-type and mutant *SOD1* mice at pre-symptomatic, symptomatic and end-stage time points revealed that upregulation of *Iba1*, *Tyrobp* and *Trem2* at the pre-symptomatic phase triggers microgliosis and the formation of the phagocytic microglial phenotype in this ALS model [13]. Transcriptome analysis of microglia from *SOD1^G93A^* mice revealed two major gene clusters. Cluster 1 was related to suppressed homeostatic genes including *P2ry12*, *Tmem119*, *Gpr34*, *Jun*, *Olfml3*, *Csf1r*, *Hexb*, *Mertk*, *Rhob*, *Cx3Cr1*, *Tgfbr1* and *Tgfb1* and transcription factors such as *Mef2a*, *Mafb*, *Jun*, *Sall1* and *Egr1*, which were enriched in adult microglia; and Cluster 2 was related to upregulated genes including *Spp1*, *Itgax*, *Axl*, *Lilrb4*, *Clec7a*, *Ccl2*, *Csf1* and *Apoe* [82]. Microglia from *SOD1^G93A^* spinal cords showed a negative correlation in the expression levels of *Mef2a*, *Sall1* and *Tgfbr1* but a positive correlation in *Apoe* expression with the progression of disease [82]. In the microglia of *SOD1^G93A^Trem^−/−^* mice, 11 out of 36 downregulated inflammatory genes were identified, and in the microglia of *SOD1^G93A^Trem^+/−^* mice 66 out of 240 suppressed homeostatic genes were identified [82]. Clec7a^+^ P2ry12^-^ microglia were detected in the spinal cord of *SOD1^G93A^Trem^+/−^* mice at P115, whereas Clec7a^-^ P2ry12^+^ homeostatic microglia were detected in the *SOD1^G93A^Trem^−/−^* mice [82]. Male *SOD1^G93A^Trem^+/−^* mice showed *Apoe* induction; however, *Trem2* deletion in these males suppressed *Apoe* and restored the homeostatic microglial genes including *Spi1* (*PU.1*), *Smad3*, *Tgfbr1* and *Sall1*, when compared to females through RNAseq analysis [82]. Additionally, miR-155 expression was not detected in *SOD1^G93A^ Trem^−/−^* mice [82]. miR-155 was found to be increased in the spinal cord of the pre-symptomatic and symptomatic *SOD1^G93A^* mice; however, miR-125b, miR-146a, miR-21 and miR-124 were increased only in the symptomatic *SOD1^G93A^* mice [11]. Gene ontology enrichment and network analysis of *SOD1^G93A^* microglia revealed transcriptional differences in the genes responsible for the regulation of the immune response (for example, upregulation of *Ccl5* and *Cxcl13*, downregulation of α-synuclein), chemotaxis (for example, upregulation of *Spp1*), DNA damage (for example, upregulation of *Brca1*, *P21*, *Pc*na and *Stat1* and downregulation of *Gadd45a*, *Sp3*), angiogenesis, inflammation, blood coagulation and hypoxia processes [83]. All of these mentioned changes in different CNS regions regarding microglial markers and transcriptome profiling of different *SOD1* mice is depicted in Table 2.

**Table 2:** Microglial alterations in different SOD1 animal models based on the age, tissue and method.

Model.	Strain	Age	Tissue	Molecule	Variation	Treatment	Effect after Treatment	Method	MN Survival	Ref
*SOD1^G93A^* mouse	B6SJL-Tg	4–6 weeks	spinal cord	NOS2, TLR2, TLR4, NFκB, HMGB1, IL-10, IL-18, MhcII, Cebpa, CD80, miR-125b, miR-146a, miR-21, miR-124	0	X		WB, RT-PCR		[11]
				*Nlrp3*, *Il-1β*, *Il-6*, *Tnfα*, *Arg1*, *Fizz1*, *Socs1*, *Tgf-β*, *CD206*	−			RT-PCR		
				miR-155	+					
*SOD1^G93A^* mouse	B6SJL-Tg	9 and 14 weeks	spinal cord	*Nlrp3*, *Il-1β*	+	X		RT-PCR		[12]
				*Asc*, *Il-18*	0					
				IL-1β, ASC, IL-18, CAS1	+			WB		
*SOD1^G93A^* mouse	B6SJL-Tg	10 weeks	spinal cord	Tyrobp, Trem2	+	X		sc-RNA-seq		[13]
*SOD1^G93A^* mouse	B6SJL-Tg	12–14 weeks	spinal cord	*Nos2*, *Fizz1*, *Il-6*, *Il-18*	0	X		RT-PCR		[11]
				HMGB1, NFκB, p- NFκB, MhcII, Cebpa, CD80, NLRP3, IL-1β, IL-10, TNFα, TGF-β, CD206, miR-155, miR-125b, miR-146a, miR-21, miR-124	+			WB, RT-PCR		
				TLR2, TLR4, Arg1, Socs1	−					
*SOD1^G93A^* mouse	B6SJL-Tg	13 weeks	spinal cord	*Ccl5*, *Cxcl13*, *Spp1*, *Brca1*, *P21*, *Pcna*, *Stat1*	+	X		RNA-seq		[83]
				*α-synuclein*, *Gadd45a*, *Sp3*	−					
*SOD1^G93A^* mouse	B6SJL-Tg	13 and 17 weeks	spinal cord	CX3CR1, iNOS, CD86, IL-1β, TNFα, Arg1, CD206, IL-10	+	X		IF, qPCR		[76]
*SOD1^G93A^* mouse	B6SJL-Tg	14 weeks	spinal cord	*Bdnf*, *Gdnf*, *Il-1β*, *Tnfα*, *Tgf-β*, *iNOS*, *Ifn-*γ	+	X		RT-PCR		[14]
				*Il-10*	0	X				
						tempol	↓*Bdnf*, ↓*Gdnf*, ↓*Il-1β*, ↓*Tnfα*, ↓*Tgf-β*, ↔*iNOS*, ↔*Ifn-*γ, ↔*Il-10*		↑	
*SOD1^G93A^* mouse	B6SJL-Tg	14 weeks	brain stem	*Sod1*, *Camk2b*	+	X		sc-RNA-seq		[81]
				*Nav2*, *mt-Rnr2*, *1700112E06Rik*	−					
*SOD1^G93A^* mouse	B6SJL-Tg	18 weeks	spinal cord	ROS, iNOS	+			IF, WB		[18]
						KCHO-1	↓ROS, ↓iNOS		↑	
*SOD1^G93A^* mouse	B6.Cg-Tg	17 weeks	spinal cord	Arg1, CD163	0			WB		[24]
						clemastine	↑Arg1, ↑CD163		↑	
*SOD1^G93A^* mouse	B6.Cg-Tg	21 weeks	spinal cord	pSTAT3, Kv1.3, Kv1.5	+	X		IF		[70]
*SOD1^G93A^* mouse	B6.Cg-Tg	NM	spinal cord	*Spp1*, *Itgax*, *Axl*, *Lilrb4*, *Clec7a*, *Ccl2*, *Csf1*, *Apoe*	+	X		RNA-seq		[82]
*SOD1^G93A^* mouse	C57BL/6J-Tg	19–22 weeks	spinal cord	*Il-1β*, *Nlrp3*, *Asc*, *Cas1*	+	X		RT-PCR		[73]
*SOD1^H46R^* mouse	C57BL/6N-Tg	28 weeks	spinal cord	Ubiquitin	+	X		IF		[71]
*SOD1^G86R^* mouse	FVB/N	NM	brain stem	*Nox2*, *Ccl4*, *MhcII*, *Ym1*, *Tyrobp*, *Tgf-β*, *Tgf-βr1*, *Cx3Cr1*, *Hexb*, *Tmem119*	+	X		RT-PCR		[27]
*SOD1^G93A^* mouse	NM	14 weeks	spinal cord	*Il-1β*, *Tnfα*	+	X		RT-PCR		[29]
						TAK-242	↔*Il-1β*, ↓*Tnfα*		↑	
*SOD1^G93A^* mouse	NM	15 weeks	spinal cord	TLR4, CD14, COX2	+			WB		[28]
						gamisoyo-san	↓TLR4, ↓CD14, ↓COX2			
*SOD1^G93A^* mouse	NM	18 weeks	spinal cord	HuR	+	X		WB, IF	↑	[30]

Variations in the expression of the molecules between wild-type and SOD1 model are marked with the following symbols: increase (+), no change (0) and decrease (−). Variations in the expression of the molecules between non-treated and treated SOD1 animal model are marked with the following symbols: increase (↑), no change (↔) and decrease (↓). X: no treatment was administered to the SOD1 animal models. MN survival: motor neuron survival; SOD1: superoxide dismutase 1; NOS2: nitric oxide synthase 2; TLR: toll-like receptor; HMGB1: high mobility group box protein 1; NFκB: nuclear factor kappa B; IL: interleukin; MhcII: major histocompatibility complex class II; Cebpa: CCAAT/enhancer binding protein; CD: cluster of differentiation; Nlrp3: nucleotide binding domain and leucine rich repeat containing protein 3; TNFα: tumor necrosis factor alpha; Arg1: arginase 1; Fizz1: found in inflammatory zone 1; Socs1: suppressor of cytokine signaling; TGF-β: transforming growth factor beta; Asc: apoptosis associated speck-like protein containing a CARD; Cas1: caspase 1; Tyrobp: TYRO protein tyrosine kinase binding protein; Trem2: triggering receptor expressed on myeloid cells 2; p-NFκB: phosphorylated NFκB; Ccl: C-C motif chemokine ligand; Cxcl13: C-X-C motif chemokine ligand 13; Spp: secreted phosphoprotein; Brca1: breast cancer gene 1; P21: cyclin dependent kinase inhibitor 1A; Pcna: proliferating cell nuclear antigen; Stat1: signal transducer and activator of transcription 1; Gadd45a: growth arrest and DNA damage inducible protein; Sp3: specificity protein 3; Cx3Cr1: C-X3-C motif chemokine receptor 1; iNOS: inducible nitric oxide synthase; BDNF: brain derived neurotrophic factor; GDNF: glial cell derived neurotrophic factor; Ifn-γ: interferon gamma; Camk2b: calcium/calmodulin dependent protein kinase beta 2; Nav2: neuron navigator 2; mt-Rnr2: mitochondrially encoded 16S rRNA; 1700112E06Rik: leucine rich melanocyte differentiation associated protein; COX2: cyclo-oxygenase 2; ROS: reactive oxygen species; pSTAT3: phosphorylated signal transducer and activator of transcription 3; Kv1.3/Kv1.5: voltage-gated potassium channel; Itgax: integrin subunit alpha X; Axl: tyrosine protein kinase receptor UFO; Lilrb4: leukocyte immunoglobulin like receptor B4; Clec7a: c-type lectin domain containing 7A; Csf1: colony stimulating factor 1; Apoe: apolipoprotein E; NOX2: NADPH oxidase 2; Ym1: chitinase 3 like 1; TGF-βR1: TGF-β receptor 1; Hexb: hexosaminidase subunit beta; Tmem119: transmembrane protein 119; HuR: ELAV-like RNA binding protein 1; WB: Western blotting; IF: immunofluorescence staining; RT-PCR: reverse transcription polymerase chain reaction; qPCR: quantitative polymerase chain reaction; RNA-seq: RNA sequencing; sc-RNA-seq: single-cell RNA seq; NM: not mentioned.

### 2.4. Summary of the Microglial Characteristics of the SOD1 Models

The first mutation that was associated with ALS was discovered to be included in the *SOD1* gene. The effects of the mutation in this gene, which codes for a detoxification enzyme, have been the focus of many studies aiming to interpret the pathological consequences of ALS disease more comprehensively. For this purpose, animals with *SOD1^G93A^* mutation on the B6SJL background were commonly utilized. Starting from the onset of the disease, increased microgliosis, together with elevated levels of neuroinflammation and NLRP3 inflammasome activation were detected as the main changes observed in microglia. Surprisingly, upregulation of anti-inflammatory cytokines was also observed, starting from the onset of the disease in the *SOD1^G93A^* mice. Finally, neuroprotective cytokines fail to provide the neurodegenerative counterbalance that causes microglia to induce non-cell autonomous motor neuron cell death. A large number of compounds intended to counteract the excess proinflammatory functions of microglia have been extensively studied to alleviate ALS symptoms. For example, tempol administration to both pre-symptomatic and symptomatic *SOD1^G93A^* mice and KCHO administration to pre-symptomatic *SOD1^G93A^* mice on the B6SJL background enhanced motor neuron survival by inhibiting proinflammatory cytokines and acting through the ROS/NOS mechanism, respectively. Similarly, clemastine treatment of pre-symptomatic *SOD1^G93A^* mice with a B6.Cg genetic background promoted both the production of anti-inflammatory cytokines and motor neuron survival. Furthermore, TAK-242 treatment at the early symptomatic phase and GSS treatment symptomatic of *SOD1^G93A^* mice cut down the release of proinflammatory cytokines, and probably supported motor neuron survival.

**Figure 2:**
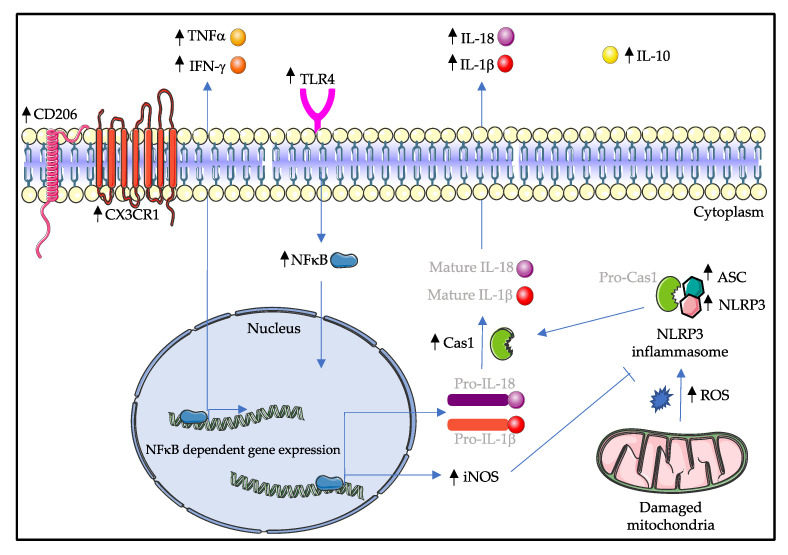
Possible signaling cascades in the spinal cord microglia of the symptomatic SOD1G93A mice. Activation of TLR4 signaling induces the translocation of NFκB into the nucleus, where NFκB can upregulate the expression of M1-type proinflammatory cytokines such as TNFα, IFN-γ, iNOS, Pro-IL-1β and Pro-IL-18. Furthermore, increased production of reactive oxygen species by damaged mitochondria induces the activation of NLRP3 inflammasome, ultimately leading to the cleavage of IL-1β and IL-18 molecules. Surprisingly, M2-type microglia markers IL-10 and CD206 are upregulated. The balance between M1 and M2 activation states is broken down in symptomatic SOD1G93A mice, and M1-type microglia outweigh M2-type microglia, causing microgliosis and eventually motor neuron degeneration. Molecules that are written in light gray are not mentioned in this review. Black arrows indicate an increase in the amount of the respective molecule. CD206: cluster of differentiation 206; CX3CR1: C-X3-C motif chemokine receptor 1; TNFα: tumor necrosis factor alpha; IFN-γ: interferon gamma; TLR4: toll-like receptor 4; IL: interleukin; NFκB: nuclear factor kappa B; Cas1: caspase 1; iNOS: inducible nitric oxide synthase; ASC: apoptosis associated speck-like protein containing a CARD; NLRP3: nucleotide binding domain and leucine rich repeat containing protein 3; ROS: reactive oxygen species.

Having now acknowledged that many proinflammatory factors are secreted by microglia, indicating increased inflammatory activity, we have outlined putative signaling pathways that could explain the characteristics found in the spinal cord of *SOD1^G93A^* mice (Figure 2).

## 3. C9Orf72 Model

A characteristic that accounts for the largest proportion of both familial and sporadic cases of human ALS in Europe is an expansion of a hexanucleotide repeat (GGGGCC) in the 1st intron of *C9Orf72* [60,84]. In Europe, approximately 30% of fALS patients and 5% of sALS patients have been identified with expansions in the *C9Orf72* gene [60]. Healthy individuals carry 2-22 repeat expansions, whereas ALS patients can have hundreds or thousands of repeats in the *C9Orf72* region [85]. Despite being the most common genetic mutation related to ALS, the function of C9Orf72 remains unclear. Several mechanisms have been proposed to describe *C9Orf72*-mediated ALS and all of them are not mutually exclusive [86]. Initial studies suggested that *C9Orf72* expansion may be a loss-of-function mutation after an approximately 40% reduction in mRNA levels of *C9Orf72* was found in the frontal cortex of ALS patients [87]. The second hypothesis is that the transcribed extensions of *C9Orf72* form nuclear RNA foci that can sequester RNA-binding proteins and disrupt RNA homeostasis [87]. Finally, the repeat-associated non-ATG (RAN) translation of hexanucleotide repeats can cause aggregation of dipeptide repeat (DPR) proteins (such as poly-GA, -GP, -GR, -PA and -PR), leading to toxicity and cellular dysfunction [88]. So far, it has been challenging to create a suitable genetically modified model with a *C9Orf72*-background that resembles human ALS. Knockout mice that exhibited a general or cell-specific loss of *C9Orf72* showed no motor or neurodegenerative abnormalities [89]. However, the same study demonstrated that *C9Orf72* was essential for the balanced functionality of microglia [89]. Since the mentioned hexanucleotide repeats are assumed to lead to translation and accumulation of DPRs, models overexpressing these have also been used. However, it was also shown that the phenotype of this model was strongly dependent on the type and frequency of expressed DPRs [31,90,91,92,93]. One of the end-stage poly-GA mouse models needed to be euthanized by 7 weeks of age, due to the severity of the symptoms [31]. In another poly-GA model, when adeno-associated virus vaccination was carried out in the pre-symptomatic stage, it was shown to be effective. Thus, ALS disease progression also differs depending on the *C9Orf72* model used, as reviewed in Batra and Lee, 2017 [94].

### 3.1. Microglial Morphology of the C9Orf72 Models

Three-week-old poly-GA mice exhibited activated amoeboid microglia in the CA2 region of the hippocampus, as shown by IBA1 staining, whereas both affected and asymptomatic poly-PR mice showed normal ramified morphology in the hippocampus region [31]. The density and the area of IBA1+ cells were reduced, together with the morphological alteration from amoeboid to ramified shape in ovalbumin-(GA)_10_-immunized poly-GA mice, when compared to control groups [33]. A promoter activity of the mouse ortholog of *C9Orf72* was detected in a few IBA1-stained microglia in the 5th layer of the primary motor cortex (15.2%), primary somatosensory cortex (16.0%), dorsal (13.2%) and the ventral (5.9%) spinal cord using *C9Orf72^Lacz+/−^* mice [95]. Immunohistochemistry and qPCR results of the spinal cord of 6-month-old poly-GA-CFP mice showed a significant upregulation of phagocytic microglia markers CD68 and IBA1 [32]. IBA1 was also found to be increased in GFP-(GR)_100_ mice on both the mRNA and protein level [34]. As a modifier of frontotemporal lobe dementia (FTLD) risk, transmembrane protein 106b (Tmem106b) was examined in *Tmem106b*+/+, +/− and *−*/*−* mice, which were injected with adeno associated virus-(GGGGCC)_66_, and which showed no statistical differences in their *Iba1* mRNA levels [96]. Similarly, mice injected with AAV1-GFP (control group), AAV1-GFP-(GA)_50_ (poly-GA group) and AAV1-GFP-(GA)_50-mut_ (disrupted poly-GA group) revealed no statistical differences in the *Iba1* mRNA levels [97].

Microglia of *C9Orf72^−^*^/*−*^ mice showed aggregation of lysotracker and Lamp1+ enlarged vesicles, together with increased levels of *Il-6* and *Il-1β* cytokines, which could support the idea that changes in lysosomal function lead to neuroinflammation [89]. Molecular changes in the microglia of different C9Orf72 models are summarized in Table 3.

### 3.2. Transcriptome Profiling of the C9Orf72 Models

RNA sequencing analysis revealed upregulation of the immune response, including cytokine/chemokine mediated signaling and interferon-inducible genes in the brain samples of end-stage poly-GA mice, of which the expressions were correlated with the spinal cord and motor cortex samples from both ALS and *C9Orf72*-ALS patients [31]. Specifically, isolated microglia from poly-GA mice showed differential expression of interferon-stimulated genes such as *Mx1*, *Isg15* and *Oasl1/2* and complement factors like *C3* and *C4b* in the transcriptome analysis [31]. Gene set enrichment analysis (GSEA) on RNA-seq samples from 17-month-old *C9Orf72^−^*^/*−*^ mice revealed that a third (6 out of 19) of the upregulated pathways were related to neuroinflammation (such as interferon signaling), in which the majority (10 out of 19) of the pathways also showed upregulation in *C9Orf72*-ALS patients [89]. Similarly, transcriptome analysis of spinal cord samples revealed that there were 233 differentially expressed genes between transgenic-PBS (TG-PBS) and ovalbumin-(GA)_10_-immunized poly-GA groups, where ovalbumin-(GA)_10_ vaccination was able to attenuate several immune pathways (for instance, induction of C-C motif chemokine ligand 4, granulin, TYRO protein tyrosine kinase binding protein and complement factors), which were triggered in poly-GA mice [33]. Genome-wide identification of RNAs from *C9Orf72* antisense oligonucleotide (ASO)- and control ASO-treated mice revealed only a few RNA expression changes in the spinal cord samples, including *C9Orf72* itself and *Cyr61* genes [98].

### 3.3. Summary of Microglial Characteristics of the C9Orf72 Models

Hexanucleotide repeat expansions in the intronic region of the *C9Orf72* gene have been described as the major genetic cause of ALS. Since its discovery, several genetically altered animal models and adeno-associated virus injection methods have been utilized to examine these expansions in detail. Surprisingly, there are only few studies investigating proinflammatory cytokines in this model. For example, interleukin release was detected in the *C9Orf72* knock-out mice. Additionally, mice overexpressing DPRs such as poly-GA and poly-GR demonstrated higher levels of microgliosis. Preferentially, studies in C9Orf72 models were mainly concentrated on transcriptome profiling. These analyses of the C9Orf72 models confirmed the upregulation of the genes related to neuroinflammation and immune pathways, especially including interferon-stimulated genes and complement factors.

## 4. TDP-43 Model

Even though mutations in transactive response DNA binding protein 43 (TARDBP or TDP-43) are rather rare in ALS patients, a mutation in about 4% of all European familial ALS cases can be found with an associated protein accumulation in the cytosol of affected cells; this process appears to play a role in the pathology of some ALS cases, although it remains unclear how these mutations cause ALS [60]. TDP-43 is a DNA and RNA binding protein, containing nuclear localization and export signals, a glycine-rich C-terminal domain and two RNA recognition motifs [99]. It plays role in RNA transcription, splicing, transport, stabilization and microRNA biogenesis [100]. TDP-43 is normally localized in the nucleus; however, under pathological conditions, it aggregates in the cytosol. The nuclear loss of TDP-43, together with its cytosolic accumulation, was shown to be involved in both ALS and FTLD pathogenesis [101]. The cleaved form of TDP-43 is the main component of the ubiquitinated and hyperphosphorylated inclusions that are present in neurons and glial cells of some ALS cases [101,102]. Until now, about 40 mutations in the *TDP-43* gene have been associated with ALS (http://www.hgmd.org, accessed in January 2021). Many of these mutations have already been introduced into an animal model to study the pathology and effects of this mutations, as reviewed in McGoldrick et al., 2013 [103]. Unfortunately, it was also found that the phenotype is strongly dependent on the genetic background of the model and the promoter used, as well as the level of the transgene expression. Thus, the findings of this animal model must still be considered with limitations [86]. Since the TDP-43 mouse models for ALS are dependent on many individual factors, it is also not easy to define a uniform course of disease, so correlation or comparison with other ALS models appears difficult.

### 4.1. Microglial Morphology of the TDP-43 Models

Increased amounts of C5aR1, the main receptor of the proinflammatory complement peptide, and C1q, one of the complement components, were demonstrated to be localized in the microglia by double immunostaining with IBA1 in 16-month-old *TDP-43^Q331K^* mice [35]. Additionally, mRNA expressions of complement components *C1qB*, *C3* and *C4* were increased; on the other hand, *CD55* was decreased both in the spinal cord samples from 10- and 16-month-old *TDP-43^Q331K^* mice, without altering C5a, *fB* and *CD59a* levels [35]. The number of activated IBA1+ microglia in *prpTDP-43^A315T^UCHL1eGFP* mice (having TDP-43 pathology in eGFP-labeled corticospinal motor neurons) increased with the disease progression, starting at P90 in the layer 2/3 and at P60 in the layer 5 of the motor cortex [36]. Additionally, affected upper motor neurons were found at close proximity to rod-like microglia [36]. Treatment of *TDP-43^A315T^* mice with root extract of anti-inflammatory *Withania somnifera* (ashwagandha) attenuated microgliosis and restored a less reactive microglia phenotype, as demonstrated in IBA1 staining [104].

The percentage of CD11b+ microglia and the number of amoeboid microglia were increased in the spinal cords of 16-month-old *TDP-43^Q331K^* mice, when compared to non-transgenic and wild-type TDP-43 mice [35]. Furthermore, female *TDP-43^A315T^* mice showed a two-fold increase in CD11b staining, and *TDP-43^A315T^SMN* (SMN: survival motor neuron) double transgenic mice attenuated the microglial activation relative to spinal cord samples of *TDP-43^A315T^* mice [37]. Chronic intraperitoneal injection of lipopolysaccharide (LPS) to heterozygous *TDP-43^A315T^* mice revealed cytoplasmic TDP-43 aggregations in LPS activated microglia, as shown by CD11b immunostaining [105].

### 4.2. Microglial Markers in the TDP-43 Models

No change in TNFα was detected in the spinal cords of ashwagandha-treated *TDP-43^A315T^* mice; however, spinal cord extracts of ashwagandha-treated *TDP-43^A315T^* mice revealed less NFκB activation [104]. On the other hand, M2-type microglia markers Ym-1 and Arginase1 were shown to be increased in ashwagandha-treated *TDP-43^A315T^* mice [104]. The molecular changes in the microglia of TDP-43 mice have been listed in Table 4.

### 4.3. Summary of the Microglial Characteristics of the TDP-43 Models

After the detection of mutations in the *TDP-43* gene of human ALS patients, TDP-43 animal models drew attention. However, studies concerning microglia in these mouse models are scarce. In one study, complement components, which drive the proinflammatory pathways, were found to be deregulated in *TDP-43^Q331K^* mice. In another study, ashwagandha treatment of *TDP-43^A315T^* mice enhanced anti-inflammatory cytokines and reduced neuroinflammation possibly by acting on the NFκB signaling pathway.

## 5. Wobbler Mouse Model

The wobbler mouse, having an autosomal recessive wobbler (*wr*) mutation, was first described by Falconer in 1956 [106] in an C57BL/Fa mouse strain. It was shown that affected C54BL/6J mice have a spontaneous mutation in the vacuolar protein sorting-associated protein 54 (*Vps54*) gene, which encodes for one of the components of the Golgi-associated retrograde protein (GARP) complex [107]. This complex plays a role in the retrograde vesicular transport of molecules from early/late endosomes to recycling endosomes and the trans-Golgi network (TGN). Nowadays, models with two different genetic backgrounds are available to study the consequences of this naturally occurring *Vps54* mutation: C57BL/6J-wr and NFR-wr. The homozygous *wr* mutation causes a destabilization of the VPS54 protein, which in turn destabilizes the GARP complex, leading to the progressive loss of upper and lower motor neurons. As the wobbler mutation occurred spontaneously, this animal model was considered for the sporadic form of ALS. In some of the ALS cases, patients were identified with the *Vps54* mutation through the Project MinE study (http://databrowser.projectmine.com, accessed in January 2021). Based on the severity of physical symptoms, wobbler mice can be grouped into three phases: (1) pre-symptomatic, (2) early clinical (evolutionary) and (3) stabilized (stable clinical). The pre-symptomatic phase lasts from birth to 3 weeks of age. During this phase, there is almost no difference between wild-type and wobbler mice in terms of clinical symptoms [108]. The early clinical phase lasts from 3 weeks to 3 months of age, when wobbler mice usually develop symptoms such as having head tremors, muscle atrophy, wobbly gait and reduced body weight [108]. The stabilized phase, which lasts from 3 months of age to death, can be identified by the arrest of motor neuron degeneration [108]. Wobbler mice at the stable clinical stage show enlarged endosome vacuolization, impaired anterograde/retrograde axonal transport, protein aggregation and mitochondrial dysfunction in the motor neurons, in addition to neuroinflammation, such as astrogliosis and microgliosis [109].

### 5.1. Microglial Morphology of Wobbler Mice

Several studies have shown neuroinflammation in different regions of the CNS of wobbler mice. The motor cortex of P20 to P60 wobbler mice showed an increased number of IBA1-labeled microglial cells [38]. P20, P40 and P60 wobbler mice also have activated microglia in all three layers of the cerebellum and white matter [39]. Increased TNFα was also demonstrated in the motor cortex and cerebellum of P40 wobbler mice by double immunostaining with IBA1 [38,39]. The percentage of amoeboid shaped IBA1+ microglia was increased in the dentate gyrus, and treatment with a glucocorticoid receptor antagonist, CORT108297, reinstated the ramified phenotype in the wobbler mice [45]. Similarly, the increased number of IBA1+ cells was significantly decreased in the spinal cord sections of wobbler mice with short-term (4 days) and long-term (21 days) treatment with glucocorticoid receptor modulator CORT113176 [46,47]. In other studies, the high density of IBA1+ cells in the spinal cords was reduced when wobbler mice were treated with neuroactive steroids progesterone [42], synthetic progestin norethindrone [42] and high-affinity progesterone receptor agonist nestorone [48], respectively. Progesterone and nestorone were also able to change the amoeboid structure of microglia to a less reactive phenotype [42,48].

Reverse transcription polymerase chain reaction (RT-PCR) analysis of *CD11b* mRNA showed an increase in the spinal cord samples of wobbler mice, which was reduced with CORT113176 treatment [46,47]. In another study, progesterone treatment was shown to decrease *CD11b* mRNA levels in the wobbler mice compared to the control group, whereas norethindrone failed to perform such an effect [42]. Wobbler mice which were treated with nestorone [48] and nonhematopoietic erythropoietin derivatives carbamylated erythropoietin (CEPO) and asialo erythropoietin (ASIALO-EPO) [44] in individual studies demonstrated lowered levels of CD11b. Increased colocalization of CD11b with TNFα [40,43] and TNFα receptor 1 (TNFR1) [40] in the cervical spinal cord of wobbler mice was reduced with treatments of glutamate release inhibitor riluzole, an approved drug against ALS [43], and a toll-like receptor 4 (TLR4) antagonist, VB3323 [43]. Surprisingly, treatment of wobbler mice with PRE-084, an agonist of endoplasmic reticulum resident receptor with chaperone-like activity (S1R), showed an increment in the number of CD11b+ cells in the white matter of the spinal cord, which was contributed to the increase in the numbers of both CD68+ (M1-type microglia) and CD206+ (M2-type microglia) cells [41].

### 5.2. Microglial Markers in the Wobbler Mouse

The expression and release of proinflammatory cytokines such as TNFα and interleukins, together with the stimulation of TLR4 signaling, are the hallmarks of M1 activated microglia. The cerebella of wobbler mice displayed significantly increased expression of TNFα at P40 and *Il-1β* at both P20 and P40 time points [39]. Spinal cord samples of wobbler mice showed an increase in *Il-1β* and *Tnfα* on the mRNA level, and treatment with Sigma-1 receptor agonist PRE-084 was not able to change this inflammatory response [41]. Similarly, treatment of wobbler mice with recombinant human TNFα-binding protein 1 (rhTBP-1) could not change TNFα and TNFR1 protein expressions [40]. In another study, wobbler mice showed upregulated *Tnfα* and inducible *NOS* (*iNos*) mRNAs, which were significantly lowered by nestorone treatment [48]. A decrease in NFκB, along with an increase in inhibitor of kappa B (IκB) α in the spinal cord samples of the nestorone administered wobbler mice, proved its anti-inflammatory effects, without affecting the TLR4 expression [48]. HMGB1, a ligand of TLR4, in the wobbler microglia was also illustrated by means of a double immunofluorescence experiment with IBA1 [46]. The increased number of HMGB1+ and TLR4+ cells in the spinal cord sections was downregulated by both short- and long-term in vivo CORT113176 treatments of wobbler mice [46,47]. mRNA levels of the proinflammatory mediators *Tnfα*, *Tnfr1*, *Tlr4*, *iNos* and p65 subunit of *NFκB* were shown to be elevated in wobbler mice, and their expressions were attenuated by short-term treatment with CORT113176 [46]. Similarly, long-term treatment of wobbler mice with CORT113176 significantly decreased the mRNA levels of *TLR4*, myeloid differentiation primary response 88 (*MyD88*), *Tnfr* and *Il-18* [47]. Increased amounts of *Tnfα*, *Tlr4* and *iNos* mRNAs were decreased by progesterone treatment, whereas norethindrone failed to perform such an effect [48]. Both progesterone and norethindrone treatment in wobbler mice increased *IκBα* mRNA levels, however only progesterone increased the mRNA level of the p65 subunit of *NFκ*B [48].

The alternative M2 microglial activation is responsible for the production of anti-inflammatory molecules. Even though the M2 activation state does not attract enough attention, when compared to the M1 activation state, M2 markers have also been investigated in several studies. It was shown that *Tgf-β* gene expression was upregulated in the cerebellum of the P40 wobbler mice [39]. Furthermore, progesterone treatment of wobbler mice increased the expression level of *Tgf-β* mRNA [42]. The molecular changes in the microglia of wobbler mice have been summarized in Table 5.

### 5.3. Summary of the Microglial Characteristics of the Wobbler Mouse

Considered as an sALS animal model, wobbler mice have a mutated *Vps54* gene. This mutation causes defects in the cellular transportation system, which eventually leads to neurobehavioral abnormalities in wobbler mice, mimicking ALS symptoms. Wobbler mice having a C57BL/6J background showed increased levels of M1-type markers in the motor cortex and cerebellum. Similarly, wobbler mice on the NFR background exhibited elevated levels of M1-type cytokines in the spinal cord. Because most studies have focused on M1-type markers, unfortunately, there are only a few studies investigating M2-type cytokines in wobbler mice. However, the histological and neurological outcomes of wobbler mice bring to mind the fact that neurodegenerative cytokines released by microglia overweigh the neuroprotective ones, which ultimately exacerbates the disease starting from the early clinical stage.

There have been several in vivo studies examining the effects of different treatments of wobbler mice on microglial function. Of these, the administration of neuroactive steroids such as progesterone, nestorone and CORT113176 diminished microgliosis and enhanced motor neuron survival in wobbler mice, acting through the NFκB signaling pathway. On the other hand, another steroid, norethindrone, failed to prevent the expression of neurodegenerative cytokines and therefore could not augment the motor neuron survival. Additionally, treatment of wobbler mice with rhTBP-1, an inhibitor of Tnfα, failed to change the expressions of either Tnfα or TNFR, even though it enhanced the motor neuron viability.

## 6. Conclusions

ALS is a fatal neurodegenerative disease. To date, numerous animal models have been generated to investigate the ALS disease in vivo. ALS is characterized by neuroinflammation in the CNS tissue of both patients and animal models. Together with motor neuron degeneration, microglia are activated in ALS and transform into an amoeboid shape. In a very simplified way, we can then refer to these processes as M1-type activation, where microglia release toxic proinflammatory agents, or M2-type activation, where microglia produce neuroprotective anti-inflammatory factors. The balance between M1 and M2 activation states is broken down during the course of ALS progression and M1-type activation can further exacerbate the disease. Many promising preclinical studies in some ALS models have shown that targeting the prevalent neuroinflammation has resulted in a slowing of symptoms, as well as reduced neurodegeneration. Unfortunately, however, transformation of these treatments into clinical trials has been unsuccessful. One possible reason for this is the strong heterogeneity of the causes of the disease. Our research has shown that of the four ALS mouse models considered here, only two models, SOD1 and wobbler, have been adequately studied with respect to neuroinflammation. Thus, the understanding of the role of microglia in the comparison between different models is still lacking. It has also been noticed that most of the studies deal with a pre-symptomatic or an early stage of onset of the disease. It must be considered that it will not be possible to treat future ALS-diagnosed patients before the onset or even directly at the onset of symptoms. These would be possible explanations for the failure of translation from preclinical to clinical investigations. Furthermore, the aspect of intracellular communication should be considered. Since all cell types of an ALS model have a genetic abnormality, affecting the functionality of these cells individually, it must be considered that the activity of microglia can also be affected by these cells of different backgrounds in different ways.

Neuroinflammation plays an essential role in all ALS patients. Due to the heterogeneity of the disease, it is mandatory to investigate the diverse roles of microglia, as reviewed by Cipollina et al., 2020, in many different models of ALS [110]. After all, this is the basis for finding an appropriate therapy for different ALS cohorts.

## Figures and Tables

**Table 1:** Morphology of microglia in the different amyotrophic lateral sclerosis (ALS) animal models based on genetic background, age, strain, collected tissue and microglial markers.

Model	Strain	Age	Tissue	Microglial Marker	Morphology	Reference
*SOD1^G93A^* mouse	B6SJL-Tg	4–6 weeks	spinal cord	IBA1, CD11b	ND	[11]
*SOD1^G93A^* mouse	B6SJL-Tg	9 and 14 weeks	spinal cord	IBA1	ND	[12]
*SOD1^G93A^* mouse	B6SJL-Tg	10 and 14 weeks	spinal cord	IBA1	ND	[13]
*SOD1^G93A^* mouse	B6SJL-Tg	12–14 weeks	spinal cord	IBA1, CD11b	Activated	[11]
*SOD1^G93A^* mouse	B6SJL-Tg	14 and 20 weeks	spinal cord	IBA1	ND	[14]
*SOD1^G93A^* mouse	B6SJL-Tg	16 and 19 weeks	medial reticular formation	IBA1, CD68	Amoeboid	[15]
*SOD1^G93A^* mouse	B6SJL-Tg	16–17 weeks	spinal cord	CD11b	ND	[16]
*SOD1^G93A^* mouse	B6SJL-Tg	17 weeks	retina, olfactory bulb	IBA1	Ramified	[17]
*SOD1^G93A^* mouse	B6SJL-Tg	18 weeks	spinal cord	IBA1	ND	[18]
*SOD1^G93A^* mouse	B6SJL-Tg	NM	spinal cord	IBA1	Activated	[19]
*SOD1^G93A^* mouse	B6SJL-Tg	NM	spinal cord	CD11b	ND	[20]
*SOD1^G93A^* mouse	B6.Cg-Tg	15 weeks	spinal cord	IBA1, CD40	Activated	[21]
*SOD1^G93A^* mouse	B6.Cg-Tg	16 weeks	spinal cord	IBA1, CD36	Activated	[22]
*SOD1^G93A^* mouse	B6.Cg-Tg	16 weeks	spinal cord	IBA1	ND	[23]
*SOD1^G93A^* mouse	B6.Cg-Tg	17 weeks	spinal cord	IBA1, CD68	ND	[24]
*SOD1^G93A^* mouse	B6.Cg-Tg	22 weeks	spinal cord	IBA1	ND	[25]
*SOD1^G93A^* mouse	B6J-Tg	16 weeks	motor cortex, spinal cord	IBA1	ND	[26]
*SOD1^G86R^* mouse	FVB/N	NM	spinal cord	IBA1	Activated	[27]
*SOD1^G93A^* mouse	NM	15 weeks	spinal cord	IBA1, CD11b	ND	[28]
*SOD1^G93A^* mouse	NM	14 weeks	spinal cord	IBA1	ND	[29]
*SOD1^G93A^* mouse	NM	18 weeks	spinal cord	IBA1	Activated	[30]
Poly-GA mouse	C57BL/6N-Tg	3 weeks	hippocampus	IBA1	Amoeboid	[31]
Poly-GA-CFP mouse	C57BL/6N-Tg	26 weeks	spinal cord	IBA1, CD68	Phagocytic	[32]
Poly-GA mouse	C57BL/6J-Tg	28 weeks	spinal cord	IBA1	Activated	[33]
Poly-PR mouse	C57BL/6N-Tg	4 and 68 weeks	hippocampus	IBA1	Ramified	[31]
GFP-(GR)_100_	C57BL/6J	6 weeks	brain	IBA1	ND	[34]
*TDP-43^Q331K^* mouse	C57BL/6J-Tg	10 and 16 months	spinal cord	IBA1	Amoeboid	[35]
*TDP-43^Q331K^* mouse	C57BL/6J-Tg	16 months	spinal cord	CD11b	Amoeboid	[35]
*prpTDP-43^A315T^ UCHL1eGFP* mouse	C57BL/6-Tg	3, 4 and 5 months	motor cortex	IBA1	Activated	[36]
*TDP-43^A315T^* mouse	C57BL/6 x CBA-Tg	4.5 months	spinal cord	CD11b	ND	[37]
*Wr* mouse	C57BL/6J-wr	P20, P40 and P60	motor cortex, cerebellum	IBA1	ND	[38,39]
*Wr* mouse	NFR-wr	P28	spinal cord	CD11b	Ramified and clusters	[40]
*Wr* mouse	NFR-wr	P42	spinal cord	CD11b	Ramified and amoeboid	[41]
*Wr* mouse	NFR-wr	P60	spinal cord	IBA1	Highly reactive	[42]
*Wr* mouse	NFR-wr	P60	spinal cord	CD11b	ND	[42]
*Wr* mouse	NFR-wr	P63	spinal cord	CD11b	Activated	[40]
*Wr* mouse	NFR-wr	P70	spinal cord	CD11b	ND	[43]
*Wr* mouse	NFR-wr	P84	spinal cord	CD11b	ND	[44]
*Wr* mouse	NFR-wr	P90	dentate gyrus	IBA1	Amoeboid	[45]
*Wr* mouse	NFR-wr	P150	spinal cord	IBA1	ND	[46,47]
*Wr* mouse	NFR-wr	P150	spinal cord	IBA1	Highly ramified	[48]
*Wr* mouse	NFR-wr	P150	spinal cord	CD11b	ND	[46,47,48]

*Wr*: Wobbler; P (number): postnatal days; IBA1: ionized calcium binding adaptor molecule 1; CD: cluster of differentiation molecule; ND: not described; NM: not mentioned.

**Table 3:** Microglial alterations in C9Orf72 mice based on the age, collected tissue and method.

Model.	Strain	Age	Tissue	Molecule	Variation	Method	Reference
Poly-GA mouse	C57BL/6	10 weeks	spinal cord	*Cyr61*	+	RNA-seq	[98]
Poly-GA mouse	C57BL/6J-Tg	28 weeks	spinal cord	*Ccl4*, *Grn*, *Tyrobp*	+	RNA-seq	[33]
Poly-GA mouse	C57BL/6N-Tg	7 weeks	brain	*Mx1*, *Isg15*, *Oasl1/2*, *C3*, *C4b*	+	RNA-seq	[31]
*C9Orf72^−/−^* mouse	C57BL/6J-Tg	N/A	spinal cord	*Il-1β*, *Il-6*	+	qRT-PCR	[89]

Variations are symbolized in terms of an increase (+) as compared to the age-matched wild type littermates. N/A: not applicable; Cyr61: cysteine rich angiogenic inducer 61; Ccl: C-C motif chemokine ligand; Grn: granulin; Tyrobp: TYRO protein tyrosine kinase binding protein; Mx1: interferon induced GTP binding protein Mx1; Isg15: interferon stimulated gene 15; Oasl1/2: 2′-5′-oligoadenylate synthase-like protein 1; C3: complement component 3; C4b: complement component 4b; IL: interleukin; RNA-seq: RNA sequencing; qRT-PCR: quantitative reverse transcription polymerase chain reaction.

**Table 4:** Microglial alterations in TDP-43 animal models based on the age, collected tissue and method.

Model.	Strain	Age	Tissue	Molecule	Variation	Method	Reference
*TDP-43^Q331K^* mouse	C57BL/6J-Tg	10 and 16 months	spinal cord	*C1qB*, *C4*, *C3*, *C5aR1*, C1q	+	RT-PCR, IF	[35]
*TDP-43^Q331K^* mouse	C57BL/6J-Tg	10 and 16 months	spinal cord	*fB*, *CD59a*, C5a	0	RT-PCR	[35]
*TDP-43^Q331K^* mouse	C57BL/6J-Tg	10 and 16 months	spinal cord	*CD55*	−	RT-PCR	[35]
*TDP-43^A315T^* mouse *	C57BL/6-Tg	N/A	spinal cord	Ym1, Arg1	+	WB	[104]
*TDP-43^A315T^* mouse *	C57BL/6-Tg	N/A	spinal cord	Tnfα	0	WB	[104]
*TDP-43^A315T^* mouse *	C57BL/6-Tg	N/A	spinal cord	p65 of NFκB	−	WB	[104]

Variations are symbolized in terms of increase (+), no change (0) and decrease (−) as compared to age-matched wild-type littermates. (* In this study, vehicle treated *TDP-43^A315T^* mice were compared to ashwagandha-treated *TDP-43^A315T^* mice.) N/A: not applicable; C1q: complement component 1q; C3: complement component 3; C4: complement component 4; C1qB: C1q beta polypeptide; C5a: complement component 5a; C5aR1: C5a receptor 1; fB: complement factor B; CD59a: complement regulator; CD55: complement regulator; Ym1: chitinase 3 like 1; Arg1: arginase 1; Tnfα: tumor necrosis factor alpha; NFκB: nuclear factor kappa B; RT-PCR: reverse transcription polymerase chain reaction, IF: immunofluorescent staining; WB: Western blotting.

**Table 5:** Microglial alterations in wobbler mice based on the age, collected tissue and method.

Model.	Strain	Age	Tissue	Molecule	Variation	Treatment	Molecule after Treatment	Method	MN Survival	Ref
*Wr* mouse	C57BL/6J-wr	P40	motor cortex	TNFα, Cas3	+	X		IF		[38]
*Wr* mouse	C57BL/6J-wr	P40	cerebellum	*Tnfα*, *Tgf-β*	+	X		RT-PCR		[39]
*Wr* mouse	C57BL/6J-wr	P20 and P40	cerebellum	*Il-1β*, *Il-10*	+	X		RT-PCR		[39]
*Wr* mouse	NFR/wr	P28	spinal cord	TNFα, CD11b	+	X		IF		[40]
*Wr* mouse	NFR/wr	P63	spinal cord	TNFα, TNFR,	+			IF		[40]
						rhTBP-1	↔TNFα, ↔TNFR, ↓CD11b		↑	
*Wr* mouse	NFR/wr	P70	spinal cord	TNFα	+			IF		[43]
						riluzole	↓TNFα, ↓CD11b		↑	
*Wr* mouse	NFR/wr	P70	spinal cord	TNFα	+			IF		[43]
						VB3323	↓TNFα, ↓CD11b		↑	
*Wr* mouse	NFR/wr	P84	spinal cord	*TNFα*, *IL-1β*	+			IF, RT-PCR		[41]
						PRE-084	↔*TNFα*, ↔*IL-1β*, ↑CD68, ↑CD206		↑	
*Wr* mouse	NFR/wr	P150	spinal cord	HMGB1, TLR4, IBA1, *CD11b*, *TNFα*, *TNFR*, *iNOS*, p65 of *NFκB*	+			IHC, RT-PCR		[46]
						CORT113176	↓HMGB1, ↓TLR4, ↓IBA1, ↓*CD11b*, ↓*TNFα*, ↓*TNFR*, ↓*iNOS*, ↓p65 of *NFκB*		↑	
*Wr* mouse	NFR/wr	P150	spinal cord	IBA1, *CD11b*, *TNFα*, *TLR4*, *iNOS*	+			IHC, RT-PCR		[42]
				*Tgf-β*, p65 of *NFκB*, RAGE	0					
				*I* *κ* *B*	−					
						progesterone	↓IBA1, ↓*CD11b*, ↔*TNFα*, ↓*TLR4*, ↓*iNOS*, ↑*Tgf-β*, ↑p65 of *NFκB*, ↔RAGE, ↑*IκB*		↑	
*Wr* mouse	NFR/wr	P150	spinal cord	IBA1, *CD11b*, *TNFα*, *TLR4*, *iNOS*	+			IHC, RT-PCR		[42]
				*Tgf-β*, p65 of *NFκB*, RAGE	0					
				*I* *κ* *B*	−					
						norethindrone	↓IBA1, ↔*CD11b*, ↑*TNFα*, ↔*TLR4*, ↑*iNOS*, ↔*Tgf-β*, ↔p65 of *NFκB*, ↔RAGE, ↑*IκB*		↔	
*Wr* mouse	NFR/wr	P150	spinal cord	HMGB1, TLR4, *MyD88*, *TNFR*, *IL-18*, IBA1, *CD11b*	+			IHC, RT-PCR		[47]
				p50 of *NFκB*	0					
						CORT113176	↓HMGB1, ↓TLR4, ↓*MyD88*, ↓*TNFR*, ↓*IL-18*, ↓IBA1, ↓CD11b, ↓ p50 of *NFκB*		↑	
*Wr* mouse	NFR/wr	P150	spinal cord	IBA1, *CD11b*, *TNFα*, *iNOS*, *NFκB*, *TLR4*	+			IHC, RT-PCR		[48]
				*I* *κ* *B*	0					
						nestorone	↓IBA1, ↓*CD11b*, ↓*TNFα*, ↓*iNOS*, ↓*NFκB*, ↔*TLR4*, ↑*IκB*		↑	

Variations in the expression of the molecules between wild-type and wobbler model are marked with the following symbols: increase (+), no change (0) and decrease (−). Variations in the expression of the molecules between non-treated and treated wobbler animals are marked with the following symbols: increase (↑), no change (↔) and decrease (↓). X: no treatment was administered to the wobbler mice animal models. MN survival: motor neuron survival; Wr: wobbler; P(number): postnatal days; TNFα: tumor necrosis factor alpha; Cas3: caspase 3; TGF-β: transforming growth factor beta; IL: interleukin; CD: cluster of differentiation; TNFR: TNFα receptor; HMGB1: high mobility group box protein 1; TLR4: toll-like receptor 4; iNOS: inducible nitric oxide synthase; NFκB: nuclear factor kappa B; IBA1: ionized calcium binding adaptor molecule 1; Myd88: myeloid differentiation primary response 88; IκB: inhibitor of kappa B; RAGE: receptor for advanced glycation end products; rhTBP-1: recombinant human TNFα binding protein 1; IF: immunofluorescence staining; RT-PCR: reverse transcription polymerase chain reaction; IHC: immunohistochemistry.

## Abbreviations

1700112E06Rik	Leucine rich melanocyte differentiation associated protein
5D4	Keratan sulfate
ALS	Amyotrophic lateral sclerosis
APOE	Apolipoprotein E
Arg1	Arginase 1
Asc	Apoptosis associated speck-like protein containing a CARD
ASIALO-EPO	Asialo erythropoietin
Axl	Tyrosine protein kinase receptor UFO
BDNF	Brain derived neurotrophic factor
Brca1	Breast cancer gene 1
C1q	Complement component 1q
C1qB	C1q beta polypeptide
C3	Complement component 3
C5a	Complement component 5a
C5aR1	C5a receptor 1
C9Orf72	Chromosome 9 open reading frame 72
Camk2b	Calcium/calmodulin dependent protein kinase beta 2
Cas	Caspase
CCL	C-C motif chemokine ligand
CD	Cluster of differentiation
CD11b	Cluster of differentiation molecule 11b
CD55	Complement regulator
CD59a	Complement regulator
Cebpa	CCAAT/enhancer binding protein
CEPO	Asialo erythropoietin
CEPO	Carbamylated erythropoietin
Clec7a	C-type lectin domain containing 7A
CNS	Central nervous system
COX2	Cyclo-oxygenase 2
Csf1	Colony stimulating factor 1
CX3CR1	C-X3-C motif chemokine receptor 1
Cyr61	Cysteine rich angiogenic inducer 61
EPO	Erythropoietin
fALS	Familial amyotrophic lateral sclerosis
fB	Complement factor B
Fizz1	Found in inflammatory zone 1
FUS	Fused in sarcoma
Gadd45a	Growth arrest and DNA damage inducible protein
GARP	Golgi-associated retrograde protein
GDNF	Glial cell line derived neurotrophic factor
Grn	Granulin
Hexb	Hexosaminidase subunit beta
HMGB1	High mobility group box protein 1
HuR	ELAV-like RNA binding protein 1
IκB	Inhibitor of kappa B
IBA1	Ionized calcium binding adaptor molecule 1
IF	Immunofluorescence staining
IFN-γ	Interferon gamma
IGF1	Insulin-like growth factor
IHC	Immunohistochemistry
IL	Interleukin
iNOS	Inducible NOS
Isg15	Interferon stimulated gene 15
Itgax	Integrin subunit alpha X
jALS	Juvenile amyotrophic lateral sclerosis
Kv1.3/Kv1.5	Voltage-gated potassium channel
Lilrb4	Leukocyte immunoglobulin like receptor B4
LPS	Lipopolysaccharide
MhcII	Major histocompatibility complex class II
mt-Rnr2	Mitochondrially encoded 16S rRNA
Mx1	Interferon induced GTP binding protein Mx1
MyD88	Myeloid differentiation primary response 88
Nav2	Neuron navigator 2
NFκB	Nuclear factor kappa B
NLRP3	Nucleotide binding domain and leucine rich repeat containing protein 3
NOS	Nitric oxide synthase
NOX2	NADPH oxidase 2
Oasl1/2	2′-5′-oligoadenylate synthase-like protein 1
p-NFκB	Phosphorylated NFκB
P21	Cyclin dependent kinase inhibitor 1A
Pcna	Proliferating cell nuclear antigen
pSTAT3	Phosphorylated signal transducer and activator of transcription 3
qRT-PCR	Quantitative RT-PCR
RAGE	Receptor for advanced glycation end products
rhTBP-1	Recombinant human TNFα binding protein 1
RNA-seq	RNA sequencing
ROS	Reactive oxygen species
RT-PCR	Reverse transcription polymerase chain reaction
S1R	Endoplasmic reticulum resident receptor with chaperone-like activity
sALS	Sporadic amyotrophic lateral sclerosis
sc-RNA-seq	Single cell RNA-seq
Socs1	Suppressor of cytokine signaling
SOD1	Superoxide dismutase 1
Sp3	Specificity protein 3
Spp	Secreted phosphoprotein
STAT1	Signal transducer and activator of transcription 1
TDP-43	Transactive response DNA protein 43
TGF-βR	TGF-β receptor
TGFβ	Transforming growth factor beta
TGN	Trans-Golgi network
TLR4	Toll-like receptor 4
Tmem119	Transmembrane protein 119
TNFα	Tumor necrosis factor alpha
TNFR1	TNFα receptor 1
Trem2	Triggering receptor expressed on myeloid cells 2
Tyrobp	TYRO protein tyrosine kinase binding protein
VPS54	Vacuolar protein sorting-associated protein 54
WB	Western blotting
Wr	Wobbler
Ym1	Chitinase 3 like 1
